# X-ray dynamical diffraction in amino acid crystals: a step towards improving structural resolution of biological molecules *via* physical phase measurements^1^

**DOI:** 10.1107/S1600576717004757

**Published:** 2017-05-08

**Authors:** Sérgio L. Morelhão, Cláudio M. R. Remédios, Guilherme A. Calligaris, Gareth Nisbet

**Affiliations:** a Instituto de Física, Universidade de São Paulo, São Paulo, SP, Brazil; b Faculdade de Física, Universidade Federal do Pará, Belém, PA, Brazil; c Institute of Physics Gleb Wataghin, University of Campinas, Campinas, SP, Brazil; d Diamond Light Source, Harwell Science and Innovation Campus, OX11 0DE, UK

**Keywords:** invariant triplet phases, multiple diffraction, d-alanine crystals, zwitterions, synchrotron X-ray diffraction

## Abstract

In this work, experimental and data analysis procedures were developed and applied for studying amino acid crystals by means of X-ray phase measurements. The results clearly demonstrated the sensitivity of invariant triplet phases to electronic charge distribution in d-alanine crystals, providing useful information for molecular dynamics studies of intermolecular forces. The feasibility of using phase measurements to investigate radiation damage mechanisms is also discussed on experimental and theoretical grounds.

## Abstract 2

X-ray phase measurements have been applied to study hydrogen bonds and radiation damage in amino acid crystals.

## 1. Introduction   

The hydrogen bond is the most important of all directional intermolecular interactions. It is ubiquitous in nature and a critical chemical bond in life science, responsible for the conformational stability of proteins and ensuring their biological functionality (Steiner, 2002 ▸; Rossi *et al.*, 2015 ▸). Within the current context of experimental and theoretical methods for molecular structure determination there are still many challenges, among them the accurate description of interactions between an electron-deficient hydrogen atom and electron-rich atoms (Reichenbächer & Popp, 2012 ▸; Tafipolsky, 2016 ▸). Particularly in protein X-ray crystallography, the detection of H atoms is one of the major problems, since they display only weak contributions to diffraction data (Ogata *et al.*, 2015 ▸). Nuclear methods such as neutron diffraction are sensitive to the proton position and combined with X-ray methods have been able to locate important H atoms to improve our understanding of macromolecular structure and function (Blakeley *et al.*, 2015 ▸). However, even in small-molecule crystals, experimental determination of electron charge in hydrogen bonds is a difficult problem, demanding charge density maps with sub-ångström resolution (Gopalan *et al.*, 2000 ▸; Krawczuk & Stadnicka, 2012 ▸).

Radiation damage in X-ray crystallography is another problem that compromises the resolution of electron density maps as well as the reliability of structure determination in biomolecules and organic samples in the crystalline state (Teng & Moffat, 2000 ▸; Blakeley *et al.*, 2015 ▸; Gerstel *et al.*, 2015 ▸; Garman & Weik, 2017 ▸). Despite all the advances in X-ray detectors and data collection protocols, radiation damage still occurs at cryogenic temperatures and the known protein structures suffer, at least to some extent, from inaccuracies originating from this effect (Pozharski *et al.*, 2013 ▸). Formation of hydrogen gas in the sample during irradiation, rather than bond cleavage, has been pointed out as the major cause for the loss of high-resolution information (Meents *et al.*, 2010 ▸). The largely incomplete understanding of the physical and chemical mechanisms behind structural damage has recently motivated the development of computational tools specifically for investigating damage creation mechanisms (Bernasconi & Brandao-Neto, 2016 ▸). In this sense, it is desirable to have an X-ray tool capable of experimentally probing small structural features such as electron charge in hydrogen bonds and radiation damage effects at atomic scales, or simply to validate high-resolution structures obtained from other experimental or purely computational methods.

### 1.1. Physical phase measurements in X-ray crystallography   

From inorganic crystals to protein crystals, structure determination with atomic resolution is mostly based on diffraction techniques (X-rays, neutrons and electrons). However, since the coherent scattering cross sections for X-rays by atoms have intermediate values between those for electrons and neutrons, physical measurements of structure factor phases have been feasible with X-rays only (Amirkhanyan *et al.*, 2014 ▸). Dynamical diffraction taking place within perfect domains is another requirement for physical phase measurements *via* multiple diffraction (MD) experiments. In crystals with small unit cells, the dynamical diffraction regime is achieved in much smaller domains than in crystals with large cells such as protein crystals: a fact that has allowed phase measurements to reveal structural details – inaccessible by other techniques – in magnetic materials (Shen *et al.*, 2006 ▸) and optical crystals with dopant ions (Morelhão *et al.*, 2011 ▸; Amirkhanyan *et al.*, 2014 ▸), and to resolve the chirality in crystals with no resonant atoms (Hümmer & Weckert, 1995 ▸; Shen *et al.*, 2000 ▸; Morelhão *et al.*, 2015 ▸).

**Figure 1:**
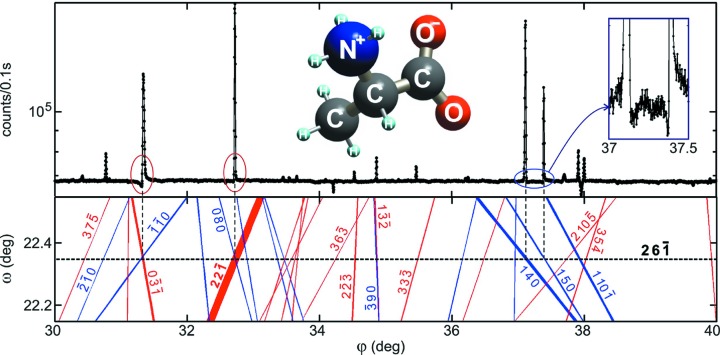
Dynamical diffraction in a d-alanine crystal, giving rise to asymmetric intensity profiles in MD cases (top panel). X-rays of 10 keV, σ polarization. Inset: d-alanine zwitterion. Graphical indexing through Bragg-cone lines (bottom panel) provides a general picture of the nearby cases, their relative strength (line thickness) and the easy distinction of the out–in/in–out geometries (blue/red lines). MD peaks are seen at the intersections of Bragg-cone lines with the 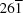 one (horizontal dashed line).

Excitation of second-order diffractions, MDs for short, and their potential applications in X-ray crystallography have been investigated since Renninger (1937 ▸) performed the first azimuthal scanning in the early 20th century, the so-called Renninger scanning. When similar experiments are carried out, very often the intensity profiles exhibit characteristic asymmetries, such as those seen in Fig. 1 ▸ (top panel), owing to dynamical coupling of the simultaneously diffracted waves inside a single-crystal domain. Over several decades, these often observed asymmetries have motivated numerous researchers in developing theoretical approaches and experimental procedures to process MD intensity profiles into structural information (Hart & Lang, 1961 ▸; Colella, 1974 ▸; Post, 1977 ▸; Chapman *et al.*, 1981 ▸; Juretschke, 1982 ▸; Chang, 1997 ▸; Weckert & Hümmer, 1997 ▸; Chang *et al.*, 1999 ▸; Wang *et al.*, 2001 ▸; Mo *et al.*, 2002 ▸; Morelhão & Kycia, 2002 ▸; Shen, 2003 ▸).

Nowadays, crystallographic studies are conducted by an increasing number of non-experts owing to substantial instrumental automation and the continuing improvement of software (Pozharski *et al.*, 2013 ▸). In this scenario, old phase measurement methods based on dynamical diffraction simulation to obtain triplet phase values within error bars are completely outdated, such that the average number of publications using this technique has dropped to less than one per year since the mid-2000s. Besides the time-consuming nature of the experiments and the need for familiarity with dynamical theory and a high level of instrumental expertise in single-crystal diffraction, the major reason discouraging further exploitation of the technique has been the low accuracy of the obtained phase values, providing no gain in structural resolution (Soares *et al.*, 2003 ▸). However, it has been well known for some time that the type of asymmetry, *i.e.* if the MD intensity profile has lower/higher (L|H) or higher/lower (H|L) shoulders, is a reliable source of information even in crystals with some mosaicity (Chang, 1984 ▸; Shen & Colella, 1986 ▸; Weckert & Hümmer, 1997 ▸; Thorkildsen *et al.*, 2003 ▸; Morelhão, 2003 ▸). Very recently, it has been proposed that this fact leads to a window of accuracy in phase measurements, implying new strategies on how to look at these asymmetries, and opening opportunities for high-resolution studies of crystal structures (Morelhão *et al.*, 2015 ▸).

In this work, to demonstrate in practice one such strategy and to highlight its potential in structural biology, we choose the challenge of detecting electron charge in hydrogen bonds responsible for intermolecular forces between amino acid molecules. The strategy is described step by step from experiment planning to data analysis procedures. Easy computer codes are used and no dynamical diffraction simulation is needed. Reliable phase information is identified by a simple graphical indexing (*e.g.* Fig. 1 ▸, bottom panel), which is also very useful for other diffraction techniques in semiconductor devices and single crystals in general (Domagała *et al.*, 2016 ▸; Nisbet *et al.*, 2015 ▸). Diffraction data from single crystals of d-alanine collected at two synchrotron facilities and with different instrumentation (flux, optics and goniometry) are presented. Model structures taking into account ionic charges are proposed and refined through comparison with experimental data, leading to an ideal model to describe X-ray diffraction by this simple amino acid molecule in terms of triplet phase invariants. According to this model, van der Waals forces between d-alanine zwitterions are also acting in the crystal structure. Moreover, within our data set, we found the first insight on the possibility of using X-ray phase measurements to study radiation damage in crystals.

## 2. Model structures   

**Figure 2:**
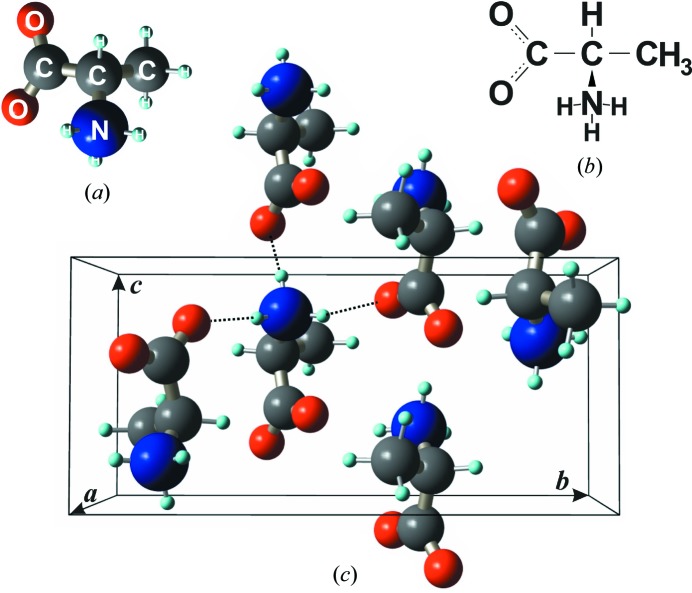
(*a*), (*b*) d-Alanine molecule, three-dimensional and flat view. (*c*) N—H⋯O bonds (dashed lines) between adjacent molecules in the crystal structure. Orthorhombic unit cell of lattice parameters *a* = 6.031 (3), *b* = 12.335 (5), *c* = 5.781 (3) Å. Twelve N—H⋯O bonds per unit cell.

With molecular formula C_3_H_7_NO_2_, l- and d-alanine are among the smallest amino acid molecules. When grown in aqueous solution, both enantiomers crystallize in space group 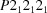 at ambient pressure, with four molecules per unit cell. The intermolecular forces are hydrogen bonds where the amine group (NH^3+^) of each molecule, in its zwitterionic form (Boldyreva, 2007 ▸; Moore *et al.*, 2011 ▸), makes N—H⋯O bonds with oxygen atoms of three carboxylate groups (COO^−^) of the nearest molecules (Fig. 2 ▸), thus linking the molecules together to form a three-dimensional crystal structure (Degtyarenko *et al.*, 2008 ▸; Funnell *et al.*, 2010 ▸). Owing to these hydrogen bonds, there is a non-spherosymmetric electron charge distribution around each amine group.

For successful use of phase measurements, the first and fundamental step in any application of this technique is the identification of MD cases susceptible to the specific structural features under investigation. This is accomplished by elaborating suitable model structures for each particular study. In our example here, we are searching for MD cases suceptible to the non-spherosymmetric electron charge distribution due to hydrogen bonds, and for this goal two simple models are initially used. One is a realistic model, denoted as the NH3 model, where the hydrogen atoms are set around the N atoms at distances of 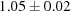
 Å (Fig. 2 ▸ *a*), as determined by neutron diffraction (Lehmann *et al.*, 1972 ▸; Wilson *et al.*, 2005 ▸). The other is a hypothetical model, denoted as the N3*e* model, where hydrogen electrons are placed in the nitrogen orbitals so that the amine group scatters X-rays as the N^3−^ ion with spherosymmetric charge distribution.

**Figure 3:**
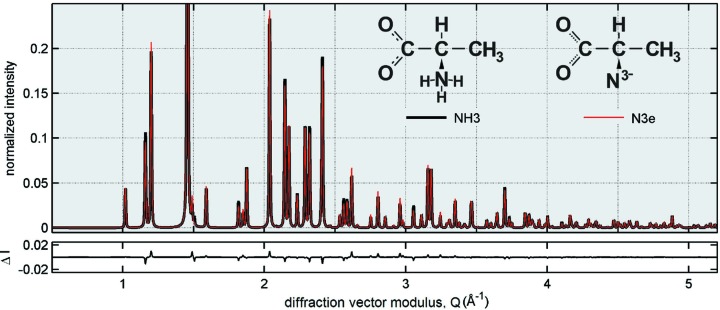
Comparison of simulated XRD patterns according to NH3 and N3*e* model structures. X-rays of 10 keV, σ polarization.

In terms of diffracted intensities, the overall differences can be seen by comparing simulated X-ray powder diffraction patterns for both models (Fig. 3 ▸). Tabulated atomic scattering factors for neutral atoms (Brown *et al.*, 2006 ▸) were used in calculating diffracted intensities of the NH3 model structure, while the atomic scattering factor of the N^3−^ ion (Morelhão *et al.*, 2015 ▸) represents the total scattering of amine groups in the N3*e* model. The comparison in Fig. 3 ▸ shows that, to distinguish between these models by such standard X-ray methods, an experimental accuracy of better than 1% (regarding the main peak) in measuring relative intensities of diffraction peaks would be required. For this reason, the realistic model NH3 is based on neutron diffraction data where no information is available on the polarization state of H atoms.

## 3. Principles of phase measurements   

Phase measurements rely on the fact that in a crystal undergoing dynamical diffraction the integrated intensity of one reflection, reflection *G*, when measured as a function of the excitation of another reflection, reflection *H*, gives rise to an intensity profile whose asymmetry depends on the triplet phase:
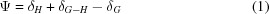
(*e.g.* Chang, 1997 ▸), where 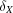 is the phase of structure factor 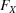 of reflection *X* (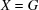, *H* and *G*–*H*).

**Figure 4:**
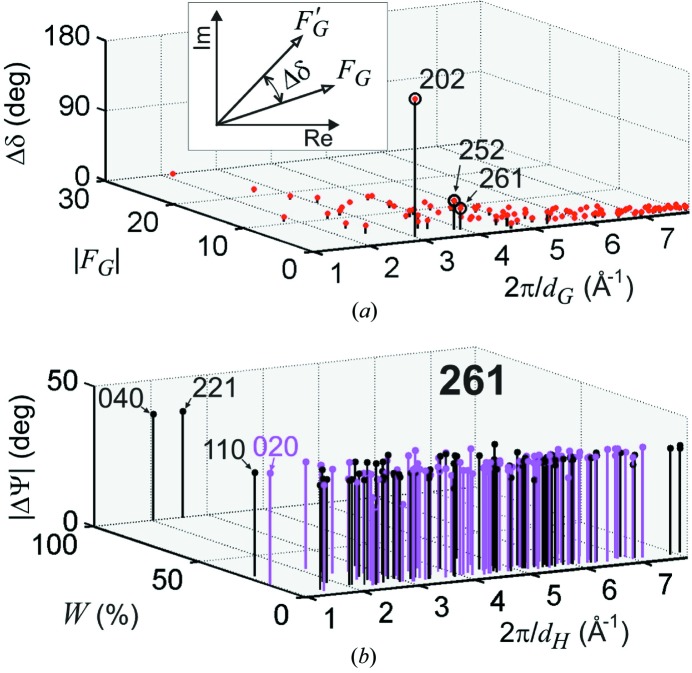
(*a*) Difference 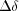 in structure factor phases regarding the proposed models, as detailed in the inset (NH3 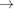 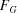 and N3*e* 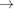 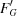). X-rays of 10 keV. (*b*) Three-beam cases predicted to show opposite profile asymmetries on each model structure according to the criterion 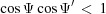. 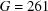 and 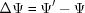 (NH3 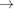 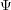 and N3*e* 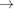 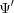). Limited to amplitude *W* > 5%. 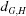 is the interplanar distance of Bragg planes.

To identify the most susceptible MD cases for studying hydrogen bonds in this amino acid crystal by phase measurements, it is necessary first to search for structure factors with phases susceptible to changes in the models, as done in Fig. 4(*a*) ▸. This indicates a few reflections, namely 202, 252 and 261, that are good candidates for phase measurements. Since these reflections have small 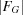 values, *i.e.* are weak reflections, they can only be used as the primary *G* reflection. After selecting the *G* reflection, it is necessary to find secondary *H* reflections that promote MD cases with opposite profile asymmetries for each of the proposed model structures. This can be done by calculating 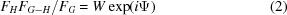
for both models and selecting the cases where the phase shift 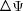 is large enough to make the triplet phase pass through the 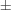
90° values, *i.e.* those cases where 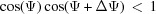. This procedure is illustrated in Fig. 4 ▸(*b*) for 261 as the *G* reflection. It predicts many cases having opposite asymmetries, including the cases for the 221 and 040 secondary reflections with the largest relative values of the amplitude *W* (see a partial list in Table 3 in §*A*3).

## 4. Graphical indexing of Renninger scans   

With a list of susceptible phases in hand, another very important step is to have an efficient method to select the most easy-to-measure MD cases capable of providing reliable phase information. A graphical indexing method based on two-dimensional representation of Bragg cones (BCs) is used here for the sake of clarity in the data analysis (§§6.1 and 6.2). For any reflection of diffraction vector 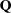, its two-dimensional BC representation is given by the relationship 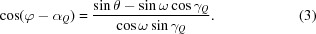
ω and φ are the instrumental angles describing the incident wavevector 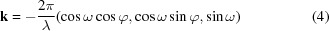
on a sample *xyz* frame where *z* is along the azimuthal rotation axis (Domagała *et al.*, 2016 ▸). The 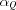 and 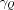 angles are obtained by projecting the diffraction vector onto this crystal frame, *i.e.*

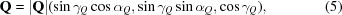
where 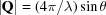 and 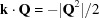.

**Figure 5:**
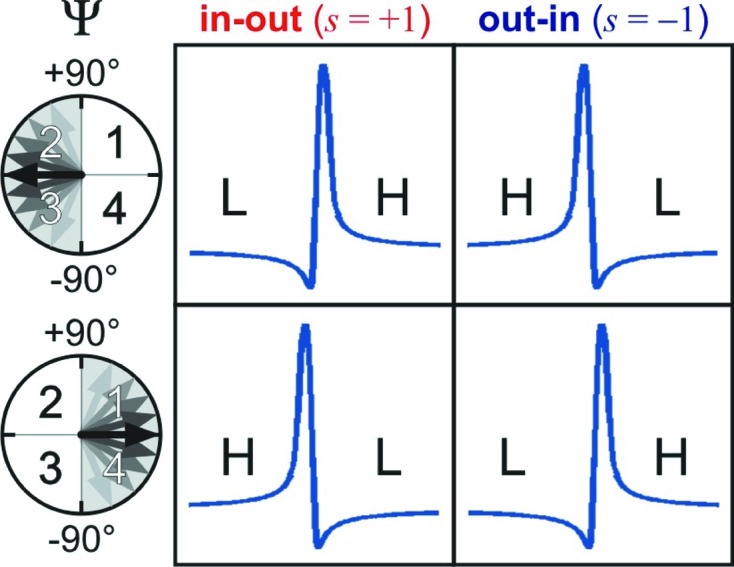
Criteria of 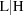 (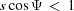) and 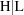 (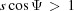) for profile asymmetry in three-beam diffraction with respect to the in–out (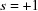) or out–in (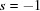) geometry of excitation and interval of values of the triplet phase Ψ: 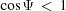, quadrants 2 and 3 (top panels), or 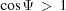, quadrants 1 and 4 (bottom panels).

Equation (3) provides two solutions for the azimuth φ as a function of the incidence angle ω. These solutions represent the two possible excitation geometries that are plotted as the out–in (blue) and in–out (red) BC lines in the 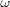
–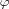 graphs, *e.g*. Fig. 1 ▸. Using lines of different colors to identify each one of these solutions is quite helpful since the observed profile asymmetries depend on both phase and excitation geometry, as summarized in Fig. 5 ▸. Another useful technique for graphically indexing Renninger scans is plotting BC lines with relative thickness. Here we use line thicknesses proportional to the amplitude *W* [equation (2)]. For instance, in the Renninger scan of reflection 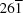 in Fig. 1 ▸, the strongest peak has the thickest BC lines owing to secondary 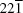 reflection.

## 5. Experimental   

Single crystals of d-alanine were grown by slow evaporation from supersaturated aqueous solutions: d-alanine powder (98% purity) diluted in distilled water, concentration of 0.25 g ml^−1^, and pH between 6 and 6.5. The solution was kept at a constant temperature (296 K) in a beaker covered with a perforated plastic lid for a period of three weeks. Transparent single crystals showing well formed natural faces were obtained, such as the one used here with approximate dimensions of 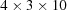
 mm and the largest face corresponding to the (130) planes (§*A*2, Fig. 12). Lattice parameters *a* = 6.031 (3), *b* = 12.335 (5) and *c* = 5.781 (3) Å were determined by X-ray powder diffraction in another sample of the same batch, and they agree very well with the MD peak positions within an accuracy of 0.01°.

X-ray data acquisition was carried out at the Brazilian Synchrotron Light Laboratory (LNLS), beamline XRD2, and at the Diamond Light Source, UK, beamline I16, testing advantages and limitations of two possible procedures for measuring line profiles of MD peaks. In one procedure, both adjustment arcs of the goniometric head are used to physically align the primary diffraction vector with one rotation axis of the sample stage. Wide azimuthal scans are possible, although eventual corrections of the incidence angle are necessary depending on the residual misalignment between the diffraction vector and the rotation axis. This procedure is preferred in terms of accuracy in both line profile and position of the peaks (Freitas *et al.*, 2007 ▸), although after fixing the sample one is limited to reflections that can be aligned within the 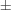
20° range of the adjustment arcs. The rotating crystal method for indexing and pre-alignment of accessible reflections has been used, as demonstrated in §*A*1.

In the other procedure, the azimuthal scans are performed by combining rotations of the diffractometer axes. This multi-axis goniometry is the standard procedure in single-crystal diffractometers. The sample is fixed at the holder within eye accuracy, and after two nonparallel reflections have been found, the crystal orientation matrix is built. With an appropriate script for azimuthal scanning, it is possible to inspect many primary reflections automatically. But, the number of accessible MD cases and the data accuracy depend on the angular range of combined rotations and the sphere of confusion of the used diffractometer.

Despite of the distinct instrumentations at the used beamlines, the energy and angular resolution were nearly the same: spectral width of 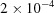 and beam divergence of 0.1 mrad. The brightness of the beam at I16 requires some attention to avoid fast radiation damage to fragile crystals stabilized by hydrogen bonds such as d-alanine. Exposure to the direct beam causes immediate damage, *e.g.* the streaks seen at the (130) surface in Fig. 12 (inset) (§*A*2). The primary 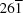 reflection was measured with the physical alignment procedure and X-rays of 10 keV, while the multi-axis goniometry procedure was used to measure a few MD cases with primaries 261 and 080, and X-rays of 8 keV. Only the primary 080 was measured in Laue transmission geometry regarding the entrance surface (130): all others in Bragg reflection geometry. The vertical scattering plane (σ polarization) was used in all measurements, where the asymmetry criteria in Fig. 5 ▸ apply for most MD cases. For other polarizations these criteria must be reviewed (Stetsko *et al.*, 2000 ▸; Morelhão & Avanci, 2001 ▸; Morelhão & Kycia, 2002 ▸).

## 6. Results and discussion   

**Figure 6:**
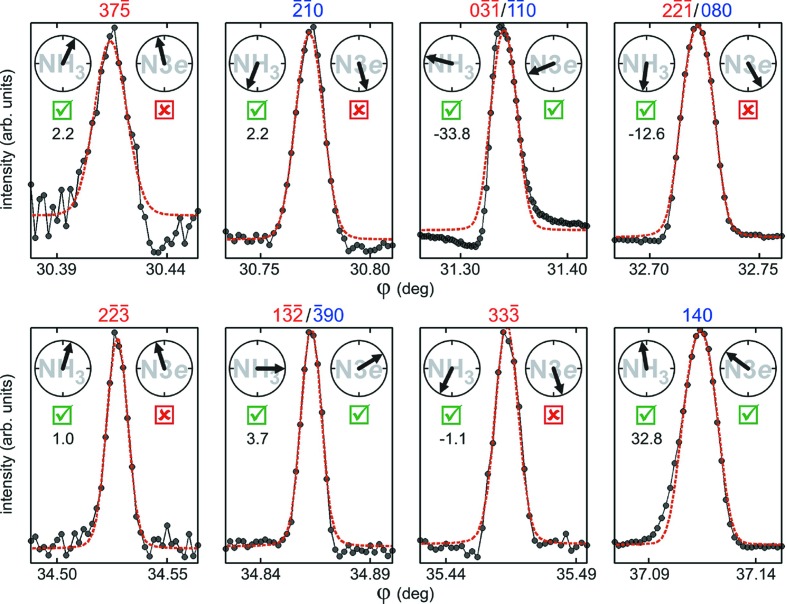
Analysis of MD peak-profile asymmetry in d-alanine. Experimental profiles (closed circles connected by lines) from Fig. 1 ▸ shown against data fitting with a symmetrical function (dashed red lines). Primary reflection 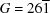. *H* reflections (blue/red indexes for out–in/in–out geometries), triplet phase values for both model structures (arrows) and their compatibility (checkbox) with the observed profile asymmetries (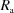 values at left) are indicated for each peak, as well as in Table 1 ▸.

Line profile asymmetries have been determined according to the value of 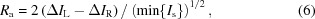
where 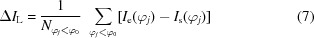
and 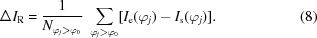


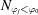
 and 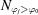 are the number of data points on the left and right side of the diffraction peak, respectively. 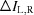 stand for the mean intensity difference on each side of the peak, since 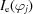 is the *j*th experimental data point and 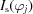 is the corresponding point obtained by data fitting with a symmetric pseudo-Voight function, which also provides the peak center 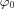. The asymmetric character of each intensity profile is therefore given as 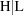 when 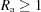 or 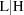 when 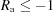. Diffraction peaks are considered symmetric, *i.e*. with an indistinguishable type of asymmetry, when 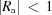. A few examples of data fitting by symmetric line profile functions are shown in Fig. 6 ▸, and their corresponding triplet phase values are given in Table 1 ▸.

Compatibility analysis between experimental asymmetries and proposed models is carried out on the basis of a true/false test according to 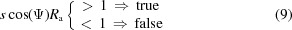
which is reliable if 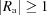. The true/false outcomes for each model are indicated by checkboxes beside each experimental profile in Fig. 6 ▸. Even profile asymmetries in the symmetric/asymmetric limit where 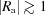 can be classified within eye accuracy, such as those at 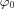 = 34.53° (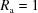) and 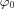 = 35.46° (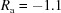). In all cases, the profile asymmetries are consistent (true) for the NH3 model only.

Let us emphasize what has been accomplished so far. By selecting just a few MD cases (Fig. 6 ▸) within a narrow Renninger scan of no more than 10°, we already demonstrate experimentally the existence of a non-spherosymmetric electron density due to H atoms around the amine group. This is an impressive result with respect to the current methods in crystallography where, to perform a similar demonstration, it would be necessary to collect thousands of reflections and solve the phase problem for constructing high-resolution electron density maps of the amine group as done by Gopalan *et al.* (2000 ▸), or to combine diffraction data and calculations of periodic density functional theory as done by Funnell *et al.* (2010 ▸). However, more refined models than NH3 are needed to explain intermolecular forces stabilizing the crystal structure, which would not exist if all atoms are neutral and unpolarized.

### 6.1. Model structure refinement   

**Figure 7:**
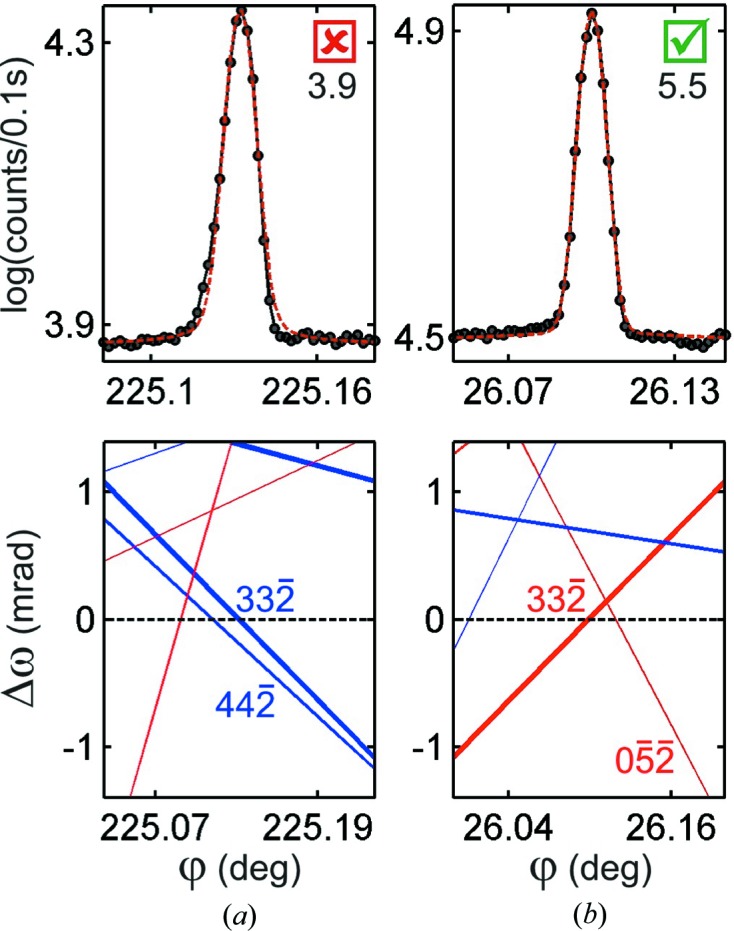
(*a*) Out–in and (*b*) in–out experimental profiles of an MD case. The 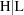 asymmetry in (*a*) is caused by the nearby 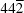 BC line. 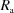 values are shown below the true/false checkbox for compatible asymmetry with the NH3 model. Horizontal dashed lines denote the 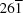 BC line.

Being able to discriminate between model structures with subtle differences is the actual challenge to phase measurements. Detecting small shifts in the triplet phases involves working with nearly symmetrical profiles whose asymmetric character can be influenced by nearby MD cases. Therefore, identification of isolated MDs is a crucial step in testing the compatibility between structure models and profile asymmetries. Coincident BC lines of comparable strength (*W* value) crossing the primary BC line at close positions can compromise the asymmetry analysis, as shown for example in Fig. 7 ▸. When the instrumentation allows the measurement of both out–in and in–out excitation geometries, as in a complete Renninger scan (§*A*2), both profiles must present opposite asymmetries. Otherwise, only the one with an isolated BC line or with very weak neighbors can be used, as in Fig. 7 ▸(*b*).

More refined models are obtained by taking into account small variations in ionic charges. To investigate the polarization of hydrogen bonds, the atomic scattering factors for the amine group are written as 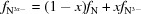 for the nitrogen and 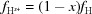 for the hydrogen atoms. 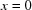 and 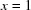 are the two extreme situations represented in the NH3 and N3*e* models, respectively. Phase measurements agree with theoretical phases for 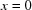. However, by slightly changing *x* we can have a more accurate idea of how susceptible the phases actually are to electron charge distribution at the amine group.

**Figure 8:**
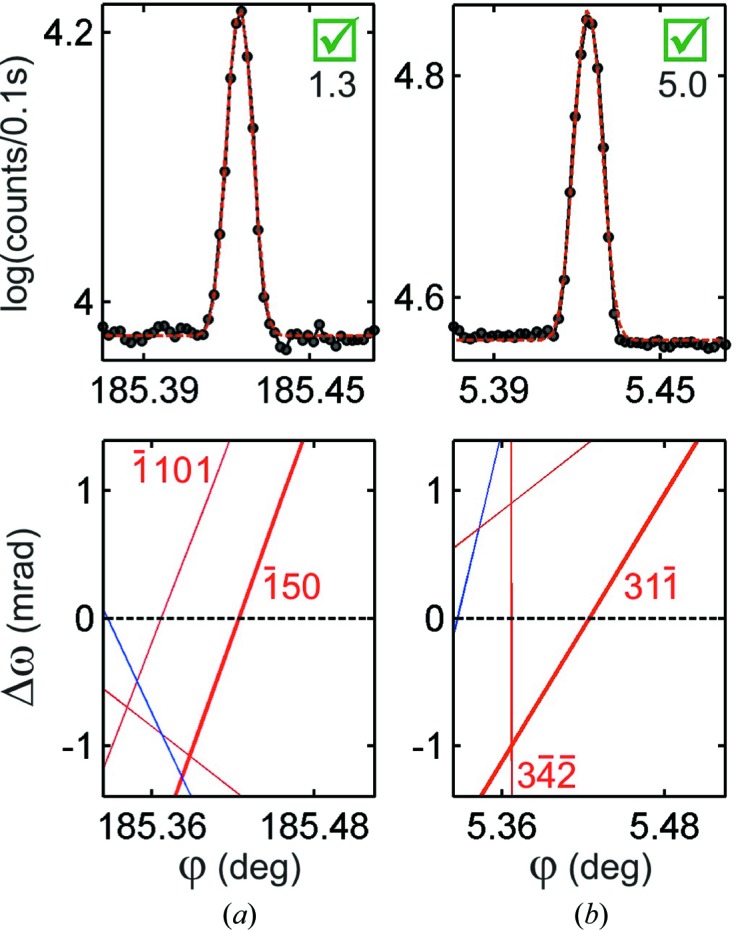
Experimental profiles and respective 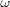
–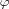 graphs of the most susceptible cases for polarization of hydrogen bonds. 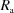 values are shown below the true/false checkbox for compatible asymmetry with the NH3 model. Horizontal dashed lines denote the 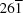 BC line.

For 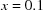, shifts in triplet phases of about 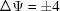
° would be enough to invert the line profile asymmetry of a few MD peaks of reasonable amplitudes 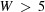
%; they are indicated as the most susceptible cases in Table 3 (§*A*3). Experimentally we are limited to the peaks with an isolated BC line and a reliable value of asymmetry (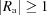) that are shown in Fig. 8 ▸. Their asymmetries are consistent with the NH3 model where 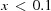, which means that H atoms in the amine group are practically neutral atoms with effective ionic charges smaller than 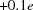.

The compatibility of other models has also been verified. Consider, for instance, a model with one electron removed from the N and shared between the O atoms. The atomic scattering factors are for the nitrogen 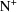 and oxygen 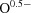 ions, while all other atoms are neutral. When compared to the NH3 model, the MD peaks that could present inversion of asymmetry are exactly the same ones previously analyzed in Fig. 8 ▸. Then, there is no evidence that the electron from the amine group is evenly shared between the two O atoms of the carboxylate group.

**Figure 9:**
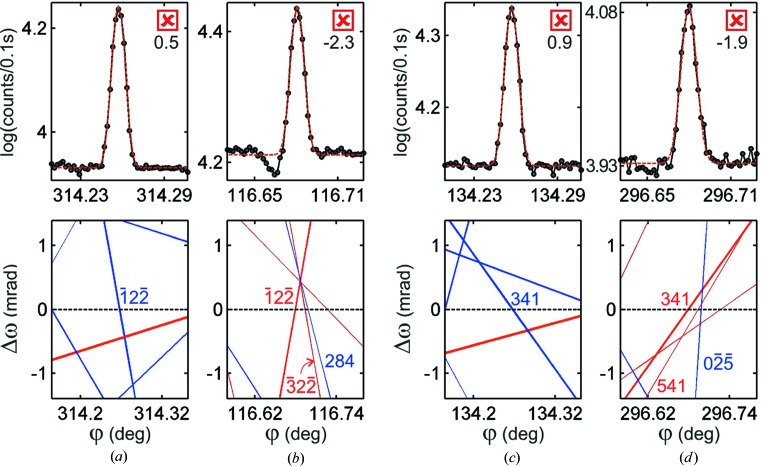
Experimental profiles and respective 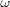
–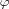 graphs of the most susceptible cases to the zwitterion model. 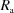 values are shown below the true/false checkbox for compatible asymmetry with the NH3 model. Horizontal dashed lines denote the 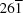 BC line.

A zwitterion model where the electron from the 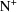 ion is placed at the nearest oxygen 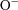 ion, as indicated in Fig. 1 (inset), seems to be compatible with the data. The discrepancies in comparison to the NH3 model are listed in Table 2 ▸ and the MD peaks of this list that could be measured are shown in Fig. 9 ▸. The phase shifts are very small and can affect only MD cases with Ψ very close to 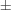
90°, whose asymmetric character is difficult to identify. Although, the four profiles agree with the zwitterion model, the most reliable profile is the one in Fig. 9 ▸(*c*), where there are no nearby BC lines and the asymmetric parameter value 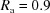 is close to the detectability limit of asymmetry established in equation (6).

### 6.2. Radiation damage   

The possibility of studying radiation damage of hydrogen bonds arises because of the high sensitivity of triplet phases to the presence of these bonds. Assuming the zwitterion model (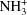
–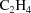
–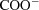) with *x* as the occupancy of H sites at N—H⋯O bonds, the MD case with 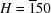 (Fig. 8 ▸ *a*) has triplet phase Ψ = −87.4° for 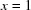 and Ψ = −93.6° for 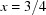 when calculated for X-rays of 8 keV. These phase values mean that phase measurements can detect one missing H atom on every four bonds or, equivalently, an average of three broken bonds per unit cell (Fig. 2 ▸ *c*).

Direct radiation damage of hydrogen bonds can be caused by Compton scattering, whose cross section for H atoms is 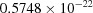
 mm^2^. To have one H^+^ ion on every four H atoms within a time scale of 10 h – a typical single-crystal experiment – the required beam flux is 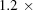 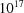
 ph mm^−2^ s^−1^, too high a value for today’s synchrotron sources. However, broken hydrogen bonds as secondary damage caused by collision of any ejected electrons from other atoms demand a much lower flux. The ionization cross section for the entire unit cell of d-alanine is 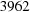 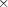 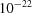
 mm^2^ when taking into account Compton and photoelectric processes (see §*A*3). Then, three ionizations per unit cell in a time period of 10 h require a flux of 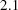 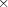 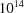
 ph mm^−2^ s^−1^. On a beam size of 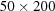
 µm, this flux corresponds to an intensity of 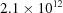
 ph s^−1^, well below the direct beam intensity of 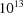
 ph s^−1^ available at the I16 beamline of the Diamond Light Source. The visible damage observed after a few seconds of exposure to such a direct beam (Fig. 12 inset, §*A*2) can be understood if each ejected electron is capable of destroying not only one but many hydrogen bonds. When the hydrogen electron is ejected either by Compton or electron collision, the H^+^ ion is repelled by the N^+^ ion, preventing any fast mechanism of electron–hole pair recombination to repair the missing bond. Formation of H_2_ gas has been reported instead (Meents *et al.*, 2010 ▸).

**Figure 10:**
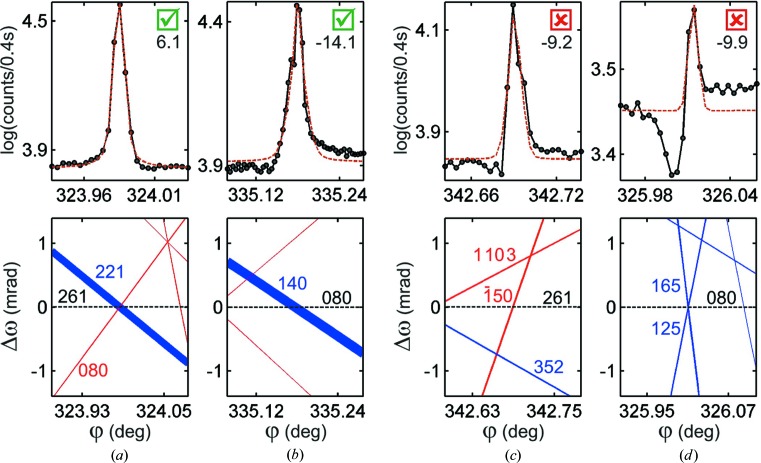
Experimental profiles and respective 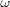
–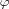 graphs of a few MD peaks measured using multi-axis goniometry for primary (*a*), (*c*) 261 and (*b*), (*d*) 080 reflections. 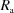 values are shown below the true/false checkbox for compatible asymmetry with the NH3 model.

**Figure 12:**
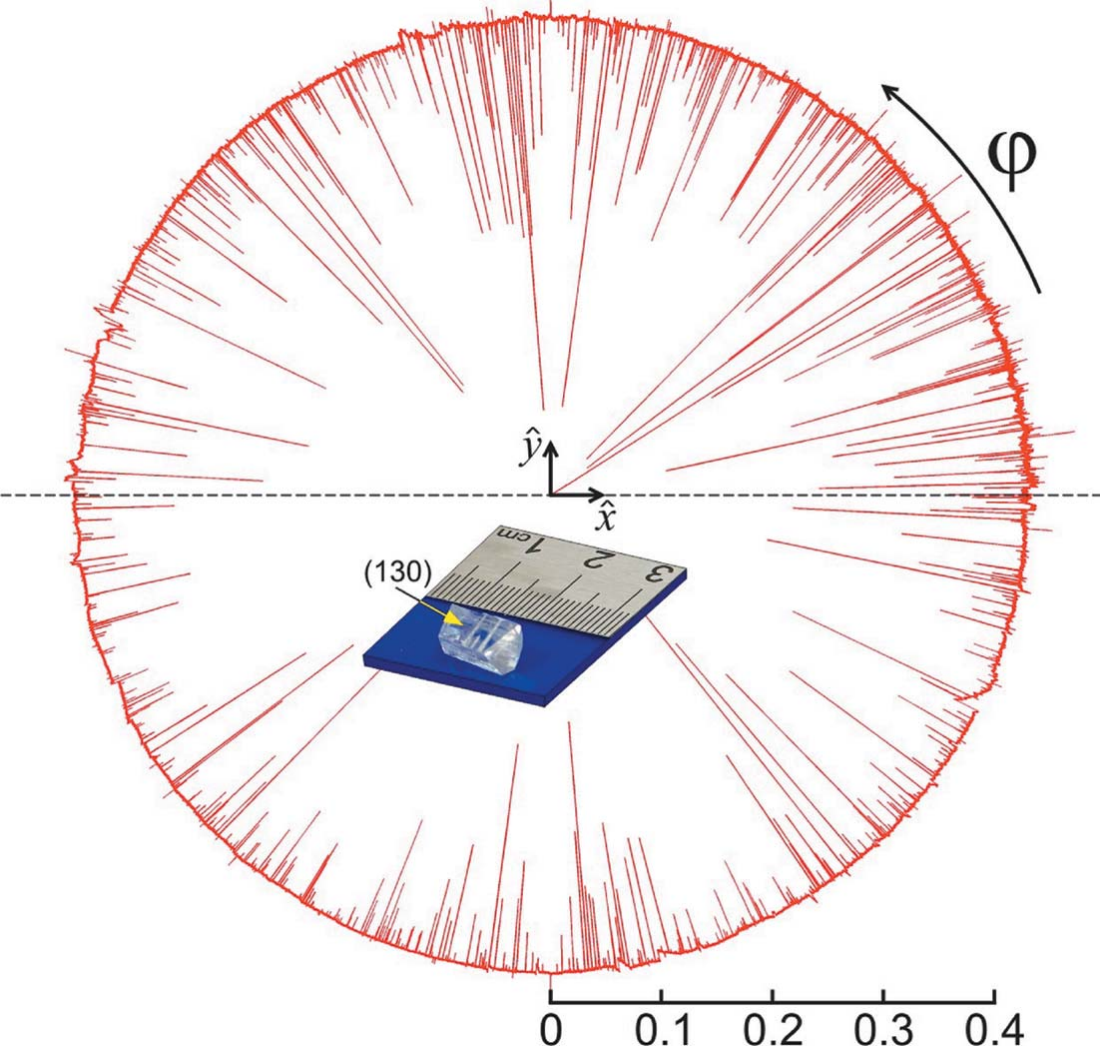
Complete Renninger scan of reflection 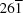 in polar plot: 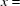 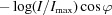 and 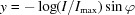. 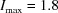
 × 10^5^ counts per 0.1 s. X-rays of 10 keV, σ polarization. Reference direction (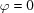): *c* axis in the incident plane pointing downstream. Sense of sample rotation: clockwise with the diffraction vector pointing to the observer. Inset: used sample showing streaks on face (130) caused by the direct beam.

With the multi-axis goniometry of beamline I16, short azimuthal scans could be performed on a few MD cases, including cases on other primary reflections. Most of the profiles agree with the zwitterion model, such as those in Figs. 10 ▸(*a*) and 10 ▸(*b*). But, there were two exceptions that are shown in Figs. 10 ▸(*c*) and 10 ▸(*d*). The MD with primary reflection 261 and secondary reflection 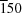 has a triplet phase very susceptible to the presence of hydrogen bonds, as discussed above. Its asymmetry, seen in Fig. 10 ▸(*c*), is clearly of the 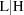 type, opposite to that seen in Fig. 8 ▸(*a*), indicating a phase shift towards a final value of 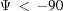
°. This shift can be explained on the basis of radiation damage when more than 25% of the intermolecular bonds have been broken during data acquisition. The direct beam was attenuated enough to avoid fast degradation of the sample due to damage on macroscopic scales that could be perceived under an optical microscope after hours of exposure. The theoretical ionization rate for such a high-intensity beam and observed phase shift are in agreement. Nevertheless, both results (theoretical and experimental) should be taken just as evidence suggesting that phase measurements are a feasible method to quantify radiation damage at the atomic level on biological single crystals. Further investigations under more controlled conditions of flux and time of exposure are still needed to delimit adequate instrumentation and procedures for this type of study.

Profile asymmetries with the primary 080 reflection are not susceptible to the subtle variations of model structures discussed in this work. All MD cases for this primary reflection should present asymmetries according to the zwitterion model. This allows us to search for MD cases that are exceptions to the asymmetry rule in Fig. 5 ▸. Although the 080 reflection diffracts under Laue transmission geometry, *i.e.* the incident and reflected beams are not on the same side of the (130) crystal surface, the only rule exception we found, shown in Fig. 10 ▸(*d*), has poor sensitivity to the triplet phase owing to polarization suppression of the second-order term of dynamical coupling responsible for the phase information (Thorkildsen & Larsen, 1998 ▸; Stetsko *et al.*, 2000 ▸; Morelhão & Kycia, 2002 ▸). This situation occurs when the *H* reflection has a Bragg angle close to 45°, such as the 125 reflection (Bragg angle of 43.9°), and diffracts in π polarization, *i.e.* its BC line appears nearly vertical in the 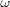
–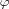 graph for the used beamline setup. Another situation compromising direct phase evaluation occurs for MD cases with very weak *Umweganregung* and strong *Aufhellung* components (Weckert & Hümmer, 1997 ▸; Rossmanith, 1999 ▸). Such cases are easily avoided when a very weak reflection can be chosen as the primary reflection. Otherwise, MD cases with very weak or polarization suppressed 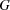
–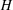 coupling reflections have poor reliability for phase measurements.

## 7. Conclusions   

The main achievement of this work is to have demonstrated in practice the full potential of phase measurements applied to current trends in crystallography. Hydrogen bonds were easily detected, a maximum value attributed to their effective polarization, model structures with subtle variations in ionic charges discriminated, relevant information for molecular dynamics studies of this amino acid crystal obtained, and insights on quantitative analysis of radiation damage discussed on theoretical and experimental grounds. Besides a model where all atoms are neutral, the only other model that can agree with the whole data set of MD profile asymmetries is the zwitterion model where an electron from the nitrogen orbitals goes to the nearest oxygen atom. In this case, the O atoms in the carboxylate group have different ionic charges and the intermolecular forces stabilizing the d-alanine crystal are also van der Waals forces between 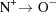 electrical dipoles. Phase sensitivity to the average number of hydrogen bonds per unit cell and experiments using high-flux synchrotron radiation point towards a damage mechanism where most of the bond cleavage is caused by photoelectron collisions. A whole package of experimental and data analysis procedures are given and explained in detail, allowing immediate use of phase measurement on a wide range of studies. The only requirements are crystals of good quality capable of undergoing dynamical diffraction and the availability of suitable structure models for each specific feature in the crystal electron density to be investigated.

## Figures and Tables

**Figure 11:**
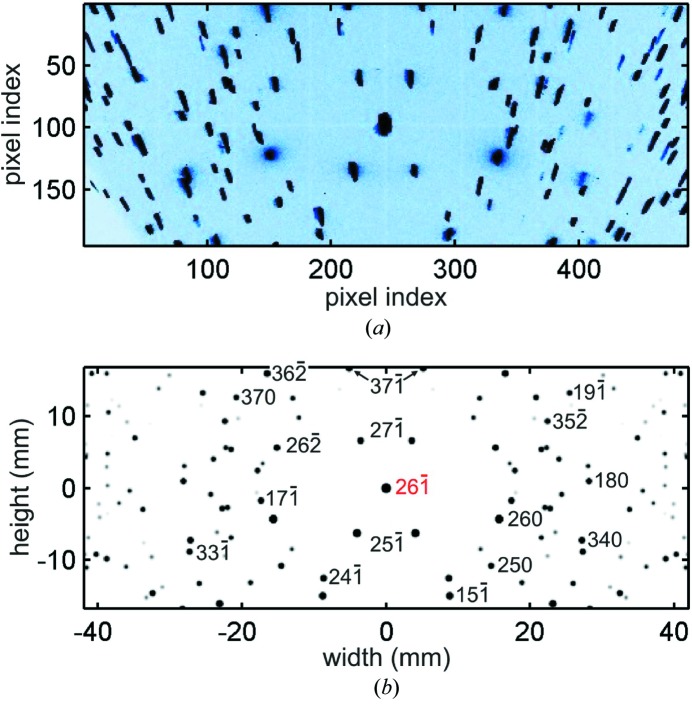
(*a*) Diffraction spots on the detector area collected in a 360° spin of the sample around the diffraction vector of reflection 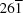. Sample–detector distance is 74.7 mm. X-rays of 10 keV, σ polarization. (*b*) Indexing of diffraction spots by simulation of the rotating crystal method [using the simulation routine in Appendix B of Morelhão (2016 ▸)]. Area detector width in horizontal direction.

**Table 1:** Theoretical triplet phases according to structure models NH3 (Ψ) and N3*e* (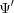) of D-alanine for a few secondary *H* reflections seen in Fig. 1 ▸ Letters b/r stand for blue/red BC lines. Experimental peak asymmetries are given in terms of parameter 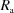, equation (6). Relative amplitudes and positions are estimated by the *W* and 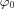 values, respectively.

*H*	Ψ (°)	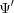 (°)	*W*(%)	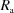		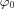 (°)
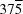 r	65	104	6	2.2	( 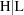 )	30.415
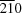 b	−111	−74	9	2.2	( 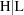 )	30.773
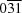 r	165	−158	29	−33.8	( 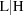 )	31.341
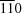 b	−3	31	16	–	–	31.341
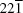 r	−98	−60	100	−12.6	( 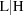 )	32.722
080b	69	106	7	—	—	32.722
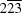 r	73	108	11	1.0	( 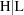 )	34.527
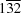 r	0	31	12	3.7	( 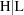 )	34.863
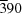 b	−111	−72	6	–	–	34.863
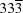 r	−116	−72	11	−1.1	( 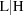 )	35.463
140b	100	143	38	32.8	( 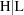 )	37.114
150b	−3	32	16	−11.5	( 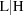 )	37.391

**Table 2:** MD cases where 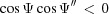 for D-alanine NH3 (Ψ) and zwitterion (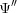) models Secondary *H* reflections diffracting at azimuth 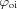 (out–in) and 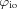 (in–out). Primary reflection 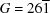. X-rays of 10 keV.

*H*	Ψ (°)	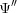 (°)	*W* (%)	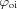 (°)	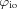 (°)
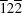	−88.8	−90.7	18	314.258	116.679
341	−88.8	−90.7	18	134.258	296.679
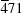	91.3	89.9	8	47.780	157.278
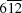	91.3	89.9	8	227.780	337.278
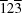	90.9	88.6	8	310.281	100.009
342	90.9	88.6	8	130.281	280.009
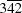	90.9	89.0	6	232.542	5.367
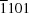	90.9	89.0	6	52.542	185.367

**Table 3:** Partial list of MD cases in which 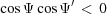 regarding the NH3 (Ψ) and N3*e* (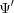) structure models of D-alanine Secondary *H* reflections diffracting at azimuth 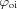 (out–in) and 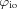 (in–out). Primary reflection 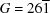. X-rays of 10 keV.

*H*	Ψ (°)	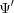 (°)	*W* (%)	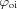 (°)	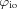 (°)
040	−98.1	−60.4	100	12.199	212.730
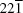	−98.1	−60.4	100	192.199	32.730
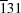	72.9	111.1	31	45.132	206.099
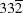	72.9	111.1	31	225.132	26.099
110	−102.3	−65.4	29	130.286	357.040
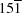	−102.3	−65.4	29	310.286	177.040
220	75.0	112.3	28	143.710	343.615
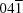	75.0	112.3	28	323.710	163.615
012	−116.0	−79.5	25	98.710	246.456
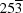	−116.0	−79.5	25	278.710	66.456
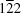	79.9	112.8	23	151.438	279.499
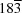	79.9	112.8	23	331.438	99.499
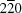	75.3	112.5	23	188.775	346.556
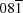	75.3	112.5	23	8.775	166.556
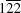	79.0	118.0	23	249.286	43.466
181	79.0	118.0	23	69.286	223.466
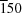 †	89.8	125.5	19	15.689	185.424
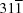 †	89.8	125.5	19	195.689	5.424
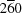	75.5	112.7	19	221.416	331.212
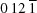	75.5	112.7	19	41.416	151.212
020	79.1	118.8	18	1.584	223.346
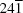	79.1	118.8	18	181.584	43.346
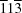 †	89.7	130.9	17	306.476	93.272
352†	89.7	130.9	17	126.476	273.272
223	73.1	107.5	17	126.720	267.779
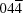	73.1	107.5	17	306.720	87.779
221	−111.9	−75.2	17	126.354	304.583
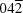	−111.9	−75.2	17	306.354	124.583
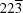	−102.6	−62.6	16	256.833	54.872
042	−102.6	−62.6	16	76.833	234.872
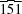	78.0	114.8	14	333.637	351.023
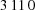	78.0	114.8	14	153.637	171.023
241	71.4	106.1	13	108.155	286.344
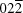	71.4	106.1	13	288.155	106.344
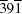	62.4	100.2	13	23.392	146.181
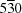	62.4	100.2	13	203.392	326.181
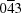	71.8	109.0	13	178.286	228.492
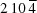	71.8	109.0	13	358.286	48.492
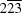	73.2	107.7	11	251.254	34.538
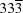	−115.7	−71.6	11	250.307	35.484
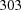 †	88.9	125.2	10	150.154	280.783
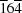 †	88.9	125.2	10	330.154	100.783
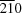	−111.1	−73.8	9	30.734	114.471
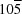 †	88.9	125.3	8	288.312	52.335
164†	88.9	125.3	8	108.312	232.335
080†	68.5	105.5	7	32.730	192.199
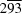 †	−91.1	−54.0	7	272.849	351.002
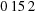 †	−91.1	−54.0	7	92.849	171.002
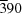	−111.3	−72.4	6	34.859	157.769
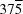	64.5	103.9	6	312.753	30.450

† Susceptible cases to polarization of hydrogen bonds.

## Appendix A

### A1. Physical alignment of primary reflection by using the rotating crystal method

With an area detector (the Pilatus 100K in our case) attached to the  arm of the single-crystal diffractometer, two-dimensional array indices  of the reference pixel receiving most of the direct beam () are identified. After fixing the sample in a goniometer head that has arcs for orientation correction, placing the area detector at a distance *D* from the sample, and moving the detector arm to a desired  value, the φ axis is spun by 360° while the detector acquires images at the rate of one frame per degree of rotation. Diffraction spots at pixels of indices *mn* correspond to reflections with scattering angles , where , ,  ,  and . In the laboratory frame,  is along the direct beam,  is vertical and  is horizontal. The pixel size for the used detector is  mm.

A diffraction spot with scattering angle close to that of the desired primary reflection is selected and aligned to the φ axis. By using as input pixel indices *mn* and azimuth  of a spot, a script suitable for the used diffractometer was written to provide the values by which each arc has to be corrected. A new spin of the rotation axis is then carried out to confirm that the aligned reflection is in fact the desired one, as shown in Fig. 11 ▸ for the  reflection.

### A2. Azimuthal scanning with a single goniometer axis

The full Renninger scan of the  reflection shown in Fig. 12 ▸ is composed of 72 uninterrupted φ scans. At the first point of each scan, the primary reflection has been centered at its rocking curve (FWHM of 0.07 mrad). The maximum drift of the rocking curve’s center as a function of φ was 0.2 mrad. In Fig. 12 ▸, smooth variations of the baseline intensity have been flattened for the sake of visualization of the 180° symmetry of the data, although the peak intensities are different since the (130) entrance surface normal direction is not the one aligned to the rotation axis.

### A3. Calculation codes for triplet phases and ionization cross sections

Structure factors taking into account non-resonant and resonant terms of the atomic scattering factors were calculated by the routine . It lists the structure factors used for comparison of phase values, as in Fig. 4 ▸ and Tables 1 ▸, 2 ▸ and 3 ▸. This routine can be found in open codes on the internet (Morelhão, 2016 ▸), as can the routines  and  used for calculating the Compton and photoelectric absorption cross sections, respectively.
